# The Role of MicroRNAs in Myeloid Cells during Graft-versus-Host Disease

**DOI:** 10.3389/fimmu.2018.00004

**Published:** 2018-01-23

**Authors:** Sophia Chen, Robert Zeiser

**Affiliations:** ^1^Department of Hematology, Oncology and Stem Cell Transplantation, Freiburg University Medical Center, Freiburg, Germany

**Keywords:** GVHD, micro RNAs, inflammation, myeloid cells, dendritic cells

## Abstract

The successful treatment of various hematologic diseases with allogeneic hematopoietic cell transplantation is often limited by the occurrence of graft-versus-host disease (GvHD). Several microRNAs (miRs) have recently been shown to impact the biology of GvHD by regulating pro- as well as anti-inflammatory target genes. There is increasing evidence that a single miR can have different effects by preferentially targeting certain genes depending on the cell type that the miR is analyzed in. This review will focus on the role of miRs in myeloid cells during the development of acute and chronic GvHD and autoimmune diseases. Because miRs act on the expression of multiple target genes and may thereby influence the immune system at different functional levels, they are potentially attractive targets for the modification of allogeneic immune responses using miR mimics and inhibitors.

## MicroRNAs (miRs)

MicroRNAs are a class of single-stranded, non-coding RNA molecules about 22 nucleotides in length that control gene expression at the post-transcriptional level (1). Initially discovered in 1993, they turned out to be important evolutionarily conserved endogenous molecules causing RNA silencing in a sequence-specific manner (2). The biogenesis of miRs is under tight temporal and spatial control and its dysregulation is associated with various diseases (2).

## The Role of miRs in Myeloid Cells During Inflammation and Autoimmunity

MicroRNAs are known to play a pivotal role in immunity, for example, by regulating toll-like receptor (TLR) signaling pathways (3). They can target multiple molecules, including TLRs themselves, their associated signaling proteins, transcription factors, and functional cytokines (3). While being crucial for physiological processes, several miRs exhibit altered expression under conditions of inflammation and autoimmune diseases.

Consistent with the fact that miR-146a controls innate immune cell and T cell responses, miR-146a was observed to be overexpressed in the peripheral blood mononuclear cells of patients with rheumatoid arthritis (4), Sjögren’s syndrome (5), and myasthenia gravis (6). Furthermore, the miR-146a single-nucleotide polymorphism (SNP) rs2910164 was linked to the development of childhood-onset asthma in Mexican patients (7), rheumatoid arthritis (8), and psoriasis (9). In addition to these descriptive findings in humans, miR-146a was also shown to function as an anti-inflammatory regulator in different cell subsets in mice (10). In dendritic cells (DCs), silencing of miR-146a and miR-146b expression was observed to enhance the production of IL-12p70, IL-6, TNF-α, and IFN-γ (11). Conversely, miR-146a overexpression in DCs was found to reduce cytokine production (11).

Another important regulatory miR in the context of immunity and inflammation is miR-155. A study reported miR-155 exerting pro-inflammatory effects in clinical and experimental arthritis, based on the observation that miR-155 was upregulated in synovial membrane and synovial fluid macrophages from rheumatoid arthritis patients (12). Mechanistically, increased expression of miR-155 in CD14^+^ monocytes was connected to reduced expression of the miR-155 target Src homology 2-containing inositol phosphatase-1 (SHIP-1) (12). Expression of SHIP-1, which is known to act as an inhibitor of inflammation, was also observed to be decreased in CD68^+^ cells in the synovial lining layer of rheumatoid arthritis patients compared to osteoarthritis patients (12). SHIP downregulation by miR-155 in myeloid cells was found in other models of inflammation as well, e.g., after infection with *Francisella tularensis* novicida (13).

## The Role of miRs in Myeloid Cells During Graft-Versus-Host Disease (GvHD)

In mouse models, GvHD biology was functionally linked to several miRs, e.g., miR-155 (14, 15), miR-100 (16), miR-146a (17, 18), miR-142 (19), miR-17-92 (20), miR-153-3p (21), and miR-29a (22).

Because miR-155 is known to regulate multiple steps in the activation of the innate immune system, a study addressed its role in DCs during GvHD in a murine model (15). By using miR-155^−/−^ bone marrow chimeric mice receiving allogeneic hematopoietic cell transplantation (allo-HCT) and miR-155^−/−^ recipient-type DCs, miR-155 deficiency in the latter cell subset was shown to cause protection from GvHD (15). Mechanistically, miR-155 deficiency led to dysregulation of the mitogen-activated protein kinase pathway and impaired DC migration toward adenosine triphosphate *in vitro* (15). In addition, activated miR-155^−/−^ DCs displayed reduced expression of different purinergic receptors and other inflammasome-associated genes (15). Functionally, caspase-1 cleavage and IL-1β production were found to be reduced in miR-155^−/−^ DCs upon activation. Consistent with the *in vitro* data, Nlrp3/miR-155 double knockout allo-HCT recipient mice displayed no increased protection from GvHD compared to Nlrp3^−/−^ recipients, indicating a functional connection between miR-155 and the Nlrp3 inflammasome (15).

While playing a crucial role in immune responses, miR-146a was also found to act as an important regulator of recipient-type DC activation during GvHD. In a murine model, miR-146a deficiency of the hematopoietic system or injection of recipient-type miR-146a^−/−^ DCs during allo-HCT was shown to cause GvHD exacerbation (18). Conversely, transfer of DCs transfected with miR-146a mimic was observed to reduce disease severity (18). Regarding the mechanism of action, absence of miR-146a in DCs increased activity of the JAK2/STAT1 pathway, leading to higher expression of class II transactivator (CIITA) and thereby causing elevated MHCII levels on the cell surface (18). Consistent with these findings, inhibition of JAK1/2 or CIITA knockdown in DCs was shown to prevent GvHD exacerbation induced by miR-146a^−/−^ DCs. At the same time, the G/C polymorphism rs2910164 within the miR-146a gene of human allo-HCT recipients, causing reduced miR-146a levels, was found to be connected to a higher risk of developing severe acute GvHD (18). The patients with said SNP in hematopoietic cells exhibited higher MHCII levels on monocytes, which could be targeted by JAK1/2 inhibition (18).

There is increasing evidence that serum or plasma miRs could be used as biomarkers and predict acute GvHD onset (23). A study suggested miR-153-3p as a potential biomarker for GvHD because miR-153-3p plasma levels were elevated on day seven after allo-HCT in the GvHD group compared to the control group even though GvHD had not occurred yet (21). Through bioinformatics analysis, indoleamine 2,3-dioxygenase (IDO) was found to be a potential target protein of miR-153-3p (21). The authors hypothesized that IDO might inhibit the development of acute GvHD through various cell types in addition to lymphocytes, such as DCs and myeloid-derived suppressor cells (21). In a previous study, IDO expression in donor precursors of plasmacytoid DCs had been shown to suppress GvHD activity of donor T cells and to influence their polarization in a murine model (24). A well-characterized subset of human myeloid-derived suppressor cells consisting of CD14^+^HLA-DR^low/neg^ cells was found to suppress T cell responses *via* IDO in patients with GvHD (25).

A recent study showed that miR-29a expression was significantly upregulated in the serum of patients at acute GvHD onset compared to patients without GvHD after allo-HCT (22). Serum miR-29a was also elevated as early as 2 weeks before time of diagnosis of acute GvHD compared to time-matched control subjects (22). In a murine model of acute GvHD, treatment with locked nucleic acid anti-miR-29a reduced mortality while retaining graft-versus-leukemia effect (22). Functionally, miR-29a was shown to activate DCs *via* TLR7 and TLR8 activation with consecutive NF-κB pathway activity and secretion of the pro-inflammatory cytokines TNF-α and IL-6 (22). Based on these different studies on miRs in GVHD this type of molecule could become a potential target for the modification of the allogeneic immune response using miR mimics and antagomirs.

Different miRs that were studied in the setting of GvHD are listed in Table 1.

**Table 1:** MicroRNAs (miRs) with a reported function in myeloid cells during graft-versus-host disease (GvHD).

miR	Functional role in myeloid cells during GvHD	Reference
miR-29a (pro-inflammatory)	miR-29a promotes inflammation through activation of DCs *via* TLR7 and TLR8	(22)
miR-146a (anti-inflammatory)	miR-146a dampens the pro-inflammatory JAK/STAT/CIITA/MHCII axis in DCs	(18)
miR-153–3p (pro-inflammatory)	miR-153-3p might enhance GvHD development by inhibiting IDO expression in different cell subsets	(21)
miR-155 (pro-inflammatory)	miR-155 is required for DC migration and Nlrp3 inflammasome activation in the GvHD context	(15)

## Author Contributions

SC and RZ contributed equally to the writing of this mini review.

## Conflict of Interest Statement

The authors declare that the research was conducted in the absence of any commercial or financial relationships that could be construed as a potential conflict of interest.
